# Case Report: Congenital simple hepatic cyst with late-onset fetal growth restriction and an unexpected neonatal phenotype

**DOI:** 10.3389/fmed.2026.1988079

**Published:** 2026-10-08

**Authors:** Claudia Marcela Angulo Seijas, Luis Enrique Castañeda Cuba, Javier Ernesto Vásquez Alvarado, Zoraida Haydee Alva Huaylla, Katherine Yolanda Lozano Peralta, Elard Ivero Jimenez Garay, Milagros Aurora Sandoval Lozano

**Affiliations:** 1 School of Medicine, Universidad Privada Antenor Orrego, Trujillo, Peru; 2 Fetal Medicine Unit, Department of Obstetrics and Gynecology, Hospital Belén de Trujillo, Trujillo, Peru

**Keywords:** case report, congenital hepatic cyst, Doppler velocimetry, fetal growth restriction, microcephaly, oligohydramnios, prenatal ultrasound

## Abstract

**Background:**

Congenital simple hepatic cysts are rare fetal abdominal lesions whose prenatal diagnosis requires differentiation from other intra-abdominal cystic abnormalities. Their coexistence with fetal growth restriction (FGR) may complicate diagnostic interpretation, particularly when additional congenital findings emerge after birth.

**Case presentation:**

A 31-year-old woman was referred at 31 + 2 weeks for third-trimester ultrasonography. A well-defined avascular cystic lesion of probable hepatic origin was identified together with severe fetal smallness and persistent oligohydramnios. Serial ultrasonography demonstrated modest cyst enlargement without biliary dilatation, mass effect, or hydrops. Severe late-onset FGR was diagnosed on the basis of an estimated fetal weight below the 3rd percentile and classified as stage I according to the Barcelona staging system in the absence of advanced Doppler abnormalities. Delivery occurred at 37 + 2 weeks. Postnatal ultrasonography confirmed a simple hepatic cyst. Microcephaly and left-hand syndactyly with digital hypoplasia were additionally identified. The infant remained asymptomatic and was managed conservatively. At 2 months, neurological examination and transfontanellar ultrasonography were normal.

**Conclusion:**

A congenital simple hepatic cyst and late-onset FGR may coexist without an established causal relationship. In this case, the unexpected postnatal identification of microcephaly and a limb anomaly broadened the differential diagnosis underlying severe fetal smallness and underscored the importance of avoiding diagnostic anchoring. Longitudinal prenatal-to-postnatal assessment enabled appropriate FGR management and conservative follow-up of an uncomplicated hepatic cyst, while the broader neonatal phenotype warrants continued developmental surveillance, genetic counseling, and diagnostic genetic testing.

## 1 Introduction

Congenital simple hepatic cysts are uncommon fetal intra-abdominal lesions. Prenatally, they typically appear as well-defined, unilocular, anechoic structures without internal vascularity, although similar sonographic features may occur in choledochal, mesenteric, enteric duplication, ovarian, and other hepatobiliary cystic lesions (1–3). Diagnostic characterization therefore relies on morphology, vascularity, anatomical relationships with the liver, portal venous system, and biliary tract, and ultimately on postnatal imaging confirmation. Most uncomplicated congenital hepatic cysts remain asymptomatic and can be managed expectantly, whereas progressive enlargement, biliary obstruction, compression of adjacent structures, or symptoms may warrant intervention (1–3).

**Table 1:** Timeline of prenatal findings, delivery, and postnatal follow-up.

Timepoint	Assessment	Key findings	Management
First trimester	Dating ultrasound	Gestational age established; no abnormality identified	Routine antenatal care
Second trimester	Anatomy scan	No cyst; fetal growth appropriate	Routine antenatal care
31 + 2 weeks	Referral ultrasound	Avascular probable hepatic cyst 19.3 × 22.1 mm; severe smallness; AFI ≈4 cm	Serial biometry, fluid and Doppler surveillance
32 + 3 weeks	Follow-up ultrasound	Cyst 18.6 × 17.3 mm; persistent oligohydramnios	Continued surveillance
36 + 3 weeks	Ultrasound and Doppler	Cyst 25.5 × 21.3 mm; EFW 1,947 g (<3rd percentile); no redistribution	Stage I FGR; delivery planned at 37 weeks
37 + 2 weeks	Repeat cesarean delivery	Female, 2,219 g; Apgar 7/8; microcephaly; left-hand syndactyly and digital hypoplasia	Multidisciplinary neonatal assessment
Postnatal day 3	Abdominal ultrasonography	Simple hepatic cyst 31.2 × 17.3 × 26.8 mm; no biliary dilatation	Conservative management
2 months	Clinical and imaging follow-up	Normal neurological and cranial ultrasound; cyst 34 × 24 mm; asymptomatic	Continued multidisciplinary follow-up

Third-trimester ultrasonography also provides an opportunity to reassess fetal growth, amniotic fluid volume, and fetoplacental hemodynamics. Late-onset FGR may be diagnosed when estimated fetal weight or abdominal circumference is below the 3rd percentile, even in the absence of abnormal Doppler findings. Although placental dysfunction is a frequent mechanism, severe late-onset fetal smallness may occasionally be the first indication of a fetal genetic or developmental disorder, particularly when the estimated fetal weight is below the 3rd percentile or additional structural findings are present. Accordingly, a normal fetoplacental Doppler profile should not preclude etiological reassessment when the phenotype broadens prenatally or after birth (5–9).

In this case, a fetal hepatic cyst was detected concurrently with severe late-onset FGR and persistent oligohydramnios. Postnatal identification of microcephaly and left-hand syndactyly with digital hypoplasia subsequently broadened the phenotype and prompted reconsideration of the etiology of the severe fetal smallness. The clinical relevance of this report therefore lies not in proposing a causal relationship between the hepatic cyst and FGR, but in illustrating how a structural fetal lesion, severe growth restriction, and an unexpected neonatal phenotype required distinct yet complementary diagnostic pathways. The prenatal-to-postnatal evolution further highlights the importance of avoiding diagnostic anchoring and reassessing the etiological interpretation of fetal growth abnormalities when additional congenital findings emerge after birth. This report was prepared in accordance with the CARE guideline.

## 2 Case description

### 2.1 Presentation and referral

A 31-year-old woman with two previous cesarean deliveries and no relevant medical history, maternal comorbidities, smoking or substance use, family history of congenital anomalies, or consanguinity was referred to our tertiary fetal medicine unit for routine third-trimester ultrasonography. Gestational age had been established by first-trimester crown-rump length measurement. The pregnancy had otherwise been uneventful, and previous examinations, including the second-trimester anatomy scan, had documented neither an intra-abdominal cystic lesion nor impaired fetal growth. Maternal blood pressure and routine antenatal laboratory findings were within normal limits.

As part of the etiological evaluation of severe fetal smallness, maternal serological testing was performed at approximately 32 weeks. Serology showed no clear evidence of acute cytomegalovirus, rubella, herpes simplex virus type 2, or parvovirus B19 infection. Toxoplasma gondii IgG was non-reactive, whereas IgM was at the lower boundary of the laboratory indeterminate range. IgG-avidity testing was not applicable in the absence of detectable IgG, and neither a repeat maternal titer nor direct infant testing was documented in the available record. Apart from microcephaly, the infant showed no rash, hepatosplenomegaly, cholestatic jaundice, or neurological abnormality, and transfontanellar ultrasonography at 2 months showed neither hydrocephalus nor intracranial calcifications. These findings did not suggest congenital toxoplasmosis, but the isolated indeterminate maternal IgM result was interpreted cautiously because the infection was not excluded by paired serology or direct infant testing.

### 2.2 Diagnostic assessment of the hepatic lesion

At 31 + 2 weeks, comprehensive ultrasonography (Mindray Resona I9 system, curvilinear 2–5 MHz transducer) identified a well-circumscribed, unilocular, anechoic cystic lesion in the upper fetal abdomen, situated to the left of the umbilical cord insertion and measuring 19.3 × 22.1 mm. Color and pulsed Doppler demonstrated no internal vascularity and no feeding vessel. The lesion abutted the intrahepatic portal venous system, moved with the liver, and was clearly separate from the stomach, gallbladder, kidneys, and bowel; the biliary tree was not dilated. There was no compression of adjacent structures, ascites, hydrops, or lesion-related hemodynamic compromise. A hepatic origin was considered most likely. At 32 + 3 weeks the lesion measured 18.6 × 17.3 mm; the small numerical difference was attributed to measurement-plane and interobserver variability. At 36 + 3 weeks it measured 25.5 × 21.3 mm while remaining avascular, thin-walled, and non-compressive (Figures 1, 2).

**Figure 1:**
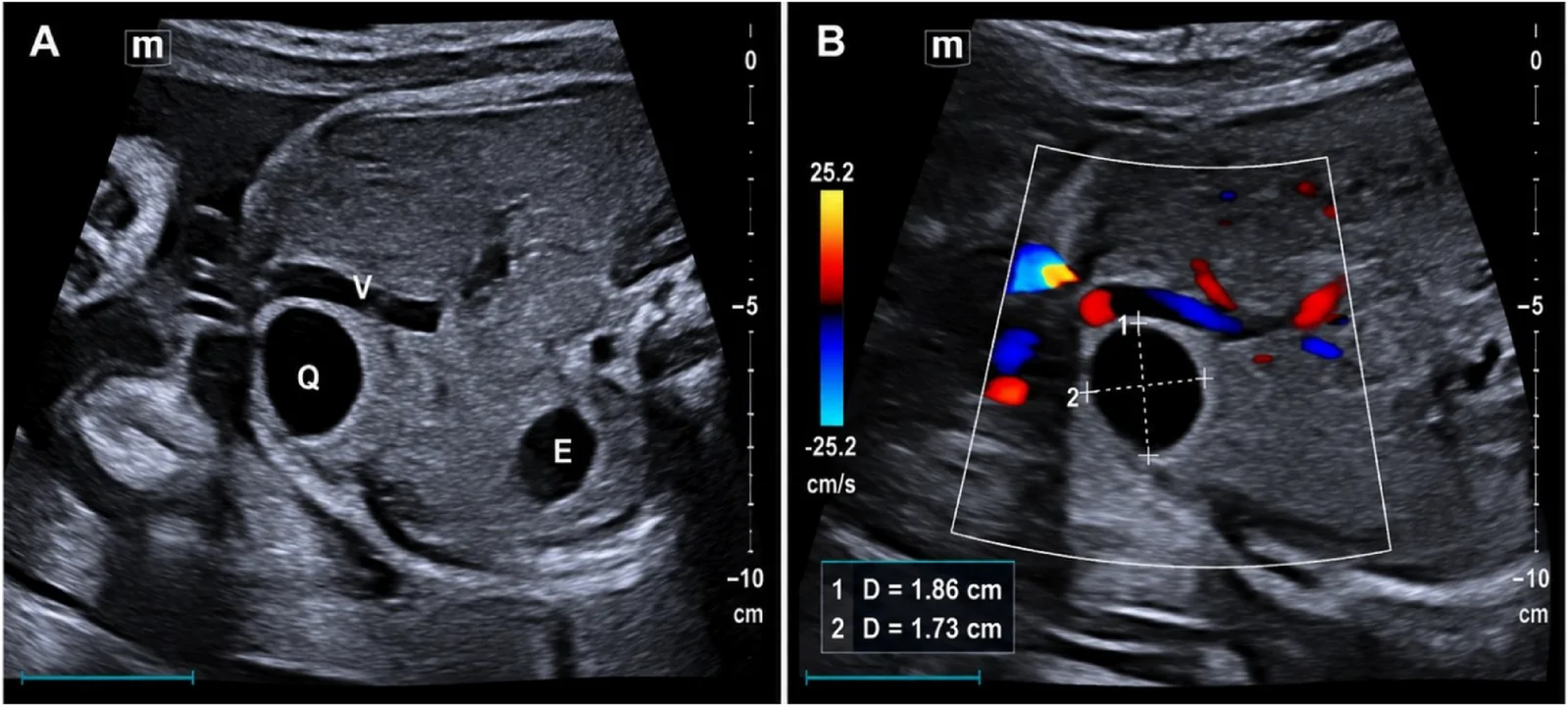
Prenatal ultrasound at 32 + 3 weeks of gestation. **(A)** Axial grayscale view of the fetal upper abdomen showing a well-defined anechoic cystic lesion (Q) of probable hepatic origin adjacent to the portal venous system (V); the stomach (E) is visualized separately. **(B)** Color Doppler demonstrates the absence of internal vascularity. The lesion measured 18.6 × 17.3 mm..

**Figure 2:**
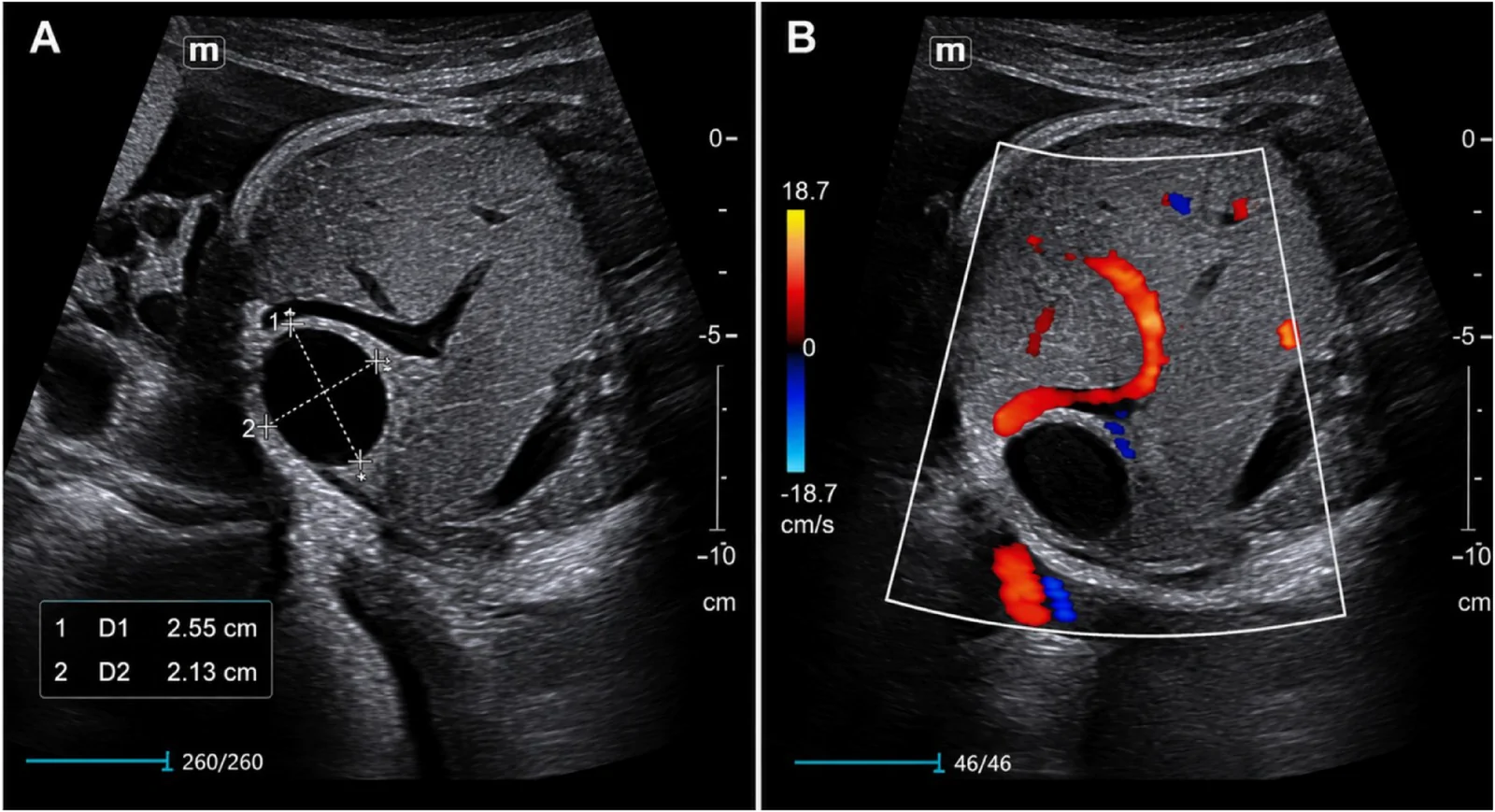
Prenatal ultrasound at 36 + 3 weeks of gestation. **(A)** Axial grayscale view demonstrating persistence of the well-defined unilocular anechoic cystic lesion, now measuring 25.5 × 21.3 mm. **(B)** Color Doppler confirms the absence of internal vascularity and demonstrates adjacent hepatic and portal vessels.

### 2.3 Fetal growth and fetoplacental Doppler

The same examination at 31 + 2 weeks disclosed severe fetal smallness, prompting serial surveillance of biometry, amniotic fluid, and fetoplacental Doppler at 1- to 2-weekly intervals. Doppler interrogation and stage classification followed the protocol of Figueras and Gratacós (6). From approximately 31 weeks, the amniotic fluid index remained approximately 4 cm. The placenta was anterior and sonographically normal. At 36 + 3 weeks the estimated fetal weight was 1,947 g (<3rd percentile); umbilical artery pulsatility index was at the 60th percentile, middle cerebral artery pulsatility index at the 99th percentile, and cerebroplacental ratio at the 83rd percentile, with umbilical artery end-diastolic flow present and no ductus venosus abnormality. Computerized cardiotocography and biophysical assessment remained reassuring.

The fetus met the Delphi consensus definition of late-onset FGR on the basis of an estimated fetal weight below the 3rd percentile (5) and was classified as stage I FGR because severe fetal smallness was present without progression to a more advanced hemodynamic stage (6). The absence of cerebral redistribution or advanced fetal hemodynamic compromise did not identify the etiology of the smallness; persistent oligohydramnios remained an additional indication for close surveillance. The timeline of prenatal findings, delivery, and postnatal follow-up is summarized in Table 1.

### 2.4 Therapeutic intervention and delivery

Given severe FGR with persistent oligohydramnios and advancing gestational age, delivery was planned at 37 + 2 weeks, consistent with the stage-based management strategy applied in this case (6, 7). The uncomplicated hepatic cyst did not influence delivery timing. An elective repeat cesarean delivery was selected because of two previous cesarean deliveries and scheduled during daytime hours to facilitate coordinated neonatal and pediatric care.

### 2.5 Neonatal findings and outcome

A female neonate weighing 2,219 g was delivered, with Apgar scores of 7 and 8 at 1 and 5 min and no need for advanced resuscitation. Head circumference was 30 cm, below −2 standard deviations for gestational age and sex according to the INTERGROWTH-21st standards (4), consistent with microcephaly. Examination revealed left-hand syndactyly with digital hypoplasia. No dysmorphic craniofacial features, cardiac murmur, hepatosplenomegaly, rash, or neurological abnormality was detected. On day 1, indirect hyperbilirubinemia associated with ABO incompatibility was treated with phototherapy for 2 days.

On postnatal day 3, abdominal ultrasonography confirmed a well-defined, unilocular, anechoic hepatic-region cyst measuring 31.2 × 17.3 × 26.8 mm, with thin walls, posterior acoustic enhancement, and no internal vascularity. The biliary ducts and hepatic parenchyma were normal. A thin layer of free fluid was noted without clinical compromise (Figure 3). Liver function tests and conjugated bilirubin were normal. Cross-sectional imaging was not performed because the sonographic features were unequivocally simple and the infant was asymptomatic.

**Figure 3:**
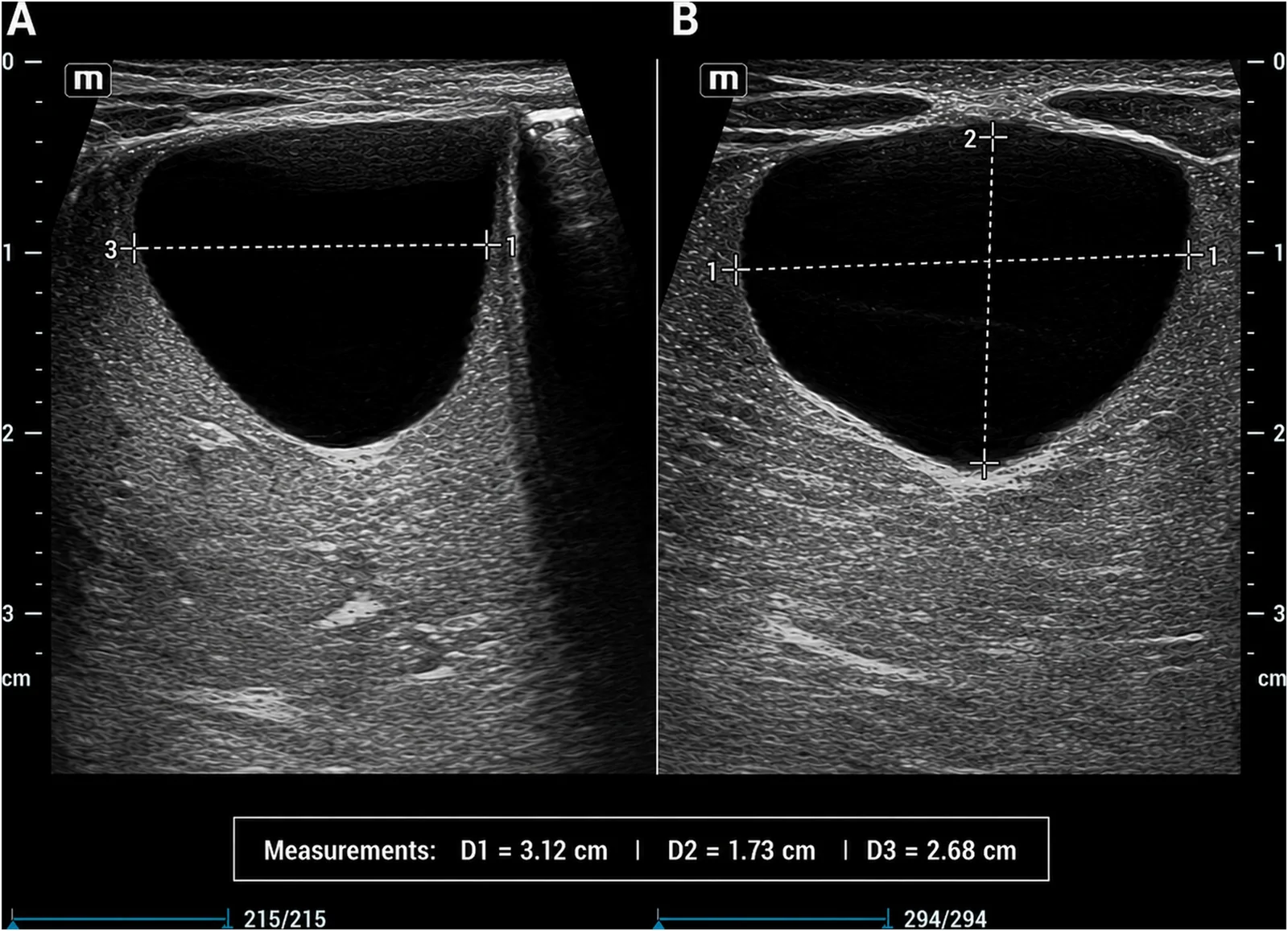
Postnatal abdominal ultrasound on day 3 confirming the congenital simple hepatic cyst. **(A)** Transverse view of the lesion, well defined, unilocular and anechoic, within the hepatic region. **(B)** Orthogonal view demonstrating the maximal transverse and anteroposterior diameters. The three orthogonal diameters obtained across both planes were 31.2 × 17.3 × 26.8 mm. The lesion has thin walls and a simple cystic appearance, with normal intrahepatic and extrahepatic bile ducts.

### 2.6 Follow-up and outcomes

The infant remained stable and asymptomatic during hospitalization. Conservative management with serial clinical and ultrasonographic follow-up was adopted. At 2 months, weight was 4,200 g, length 53 cm, and head circumference 35 cm. Neurological examination and transfontanellar ultrasonography were normal. Abdominal ultrasonography demonstrated a persistent simple anechoic cyst in hepatic segment VIII measuring 34 × 24 mm, without internal vascularity or biliary dilatation. Thyroid-stimulating hormone was within the age-specific reference range, and HSV IgM and syphilis testing were negative. Continued pediatric, imaging, growth, neurodevelopmental, and genetic follow-up was recommended.

### 2.7 Timeline

## 3 Discussion

This case illustrates the value of comprehensive third-trimester ultrasonography integrating fetal anatomy, growth, amniotic fluid volume, and fetoplacental assessment. A single examination identified both a previously unreported fetal abdominal cystic lesion and severe fetal smallness with persistent oligohydramnios, requiring complementary morphological and growth-restriction pathways.

The prenatal and postnatal imaging features were consistent with a simple congenital hepatic cyst: a well-circumscribed, unilocular, anechoic, avascular lesion that moved with the liver and remained separate from the stomach, gallbladder, bowel, and kidneys, without biliary dilatation or significant mass effect (1–3). Reported congenital hepatic cysts are commonly subcapsular or arise from the inferior/right hepatic region and may be closely related to portal or biliary structures; therefore, establishing continuity with hepatic parenchyma and demonstrating a separate gallbladder and nondilated biliary tree are more informative than abdominal laterality alone (1–3). In this patient, postnatal localization to segment VIII strengthened the hepatic diagnosis.

The maximum recorded diameter increased from 22.1 mm at 31 + 2 weeks to 34 mm at 2 months of age. Although measurements obtained in different planes and at different examinations are not strictly interchangeable, this trajectory indicates meaningful early enlargement rather than the relative stability typical of many acquired adult simple hepatic cysts. Congenital nonparasitic hepatic cysts are generally considered developmental lesions derived from aberrant intrahepatic bile-duct structures; their epithelial phenotype supports a biliary origin even when imaging shows no communication with the biliary tree and the cyst contains no bile (1–3). Their early natural history is consequently variable: enlargement, stability, spontaneous reduction, and complete regression have all been described. Proposed explanations for reduction or disappearance include involution or remodeling during maturation of the liver and biliary system, but this mechanism remains speculative. Current small case series do not support a reliable prediction that a relatively late-detected or larger fetal cyst will regress; persistence and enlargement instead justify continued imaging because intervention is driven by symptoms, obstruction, compression, or rapid progressive growth rather than size alone (1–3).

Late-onset FGR was diagnosed according to Delphi criteria, with an estimated fetal weight below the 3rd percentile fulfilling a solitary diagnostic criterion (5), and classified as stage I according to the Barcelona protocol because severe smallness occurred without advanced Doppler deterioration (6). Persistent oligohydramnios warranted close surveillance. Delivery at 37 + 2 weeks was consistent with the management strategy applied (6, 7).

The coexistence of a congenital hepatic cyst, severe FGR, microcephaly, and left-hand syndactyly with digital hypoplasia is noteworthy but does not establish causality. The small noncompressive cyst was unlikely to explain impaired growth. The postnatal phenotype raises a genetic or developmental differential despite the absence of advanced Doppler abnormalities. After clinical genetics assessment and detailed phenotyping, chromosomal microarray should be prioritized to detect pathogenic copy-number variants; if nondiagnostic, trio-based exome sequencing should follow because the combination of congenital anomalies and microcephaly may reflect a monogenic disorder (8, 9). Phenotype-directed analysis should include, among other possibilities, MYCN or MIR17HG-related Feingold syndrome, which can combine microcephaly, growth restriction, and digital anomalies, and STAMBP-related microcephaly-capillary malformation syndrome if capillary malformations, progressive microcephaly, seizures, or additional compatible features emerge. These are differential considerations rather than clinical diagnoses, because the present infant lacks several defining manifestations and definitive genetic testing has not been performed.

This broader interpretation reinforces the need to avoid diagnostic anchoring. A fetal abdominal cyst should remain a provisional anatomical diagnosis until postnatal confirmation, and biometric FGR should trigger etiological assessment rather than an automatic assumption of placental insufficiency. Additional neonatal findings can materially change that interpretation.

Strengths include longitudinal prenatal-to-postnatal characterization, serial growth and Doppler assessment, objective documentation of oligohydramnios, maternal infection screening, postnatal imaging confirmation, and multidisciplinary follow-up. Limitations include lack of histopathological confirmation, cross-sectional imaging, definitive genetic testing, placental histopathology, and long-term follow-up. Placental examination could have identified lesions of maternal or fetal vascular malperfusion supporting placental insufficiency, or conversely an absence of relevant placental disease that would have strengthened consideration of primary genetic or developmental growth restriction; however, placental histology cannot by itself exclude a genetic cause and both mechanisms may coexist (10).

## 4 Patient perspective

The mother expressed concern following prenatal identification of the abdominal cyst and impaired fetal growth. Counseling addressed the distinct implications of both findings, the rationale for surveillance and delivery planning, and the need for postnatal confirmation. Following confirmation of an uncomplicated cyst, conservative management and multidisciplinary follow-up were discussed and accepted.

## 5 Conclusion

A congenital simple hepatic cyst and late-onset FGR may coexist without an established causal relationship. The unexpected postnatal identification of microcephaly and a limb anomaly broadened the differential diagnosis underlying severe fetal smallness and underscored the importance of avoiding diagnostic anchoring. Longitudinal prenatal-to-postnatal assessment enabled appropriate FGR management and conservative follow-up of an uncomplicated hepatic cyst, while the broader phenotype warrants developmental surveillance, genetic counseling, chromosomal microarray, and, if nondiagnostic, trio exome sequencing.

## Data Availability

The original contributions presented in the study are included in the article/Supplementary Material, further inquiries can be directed to the corresponding author.
