# Supplementary material for: Case Report: Congenital simple hepatic cyst with late-onset fetal growth restriction and an unexpected neonatal phenotype
Source: Front Med (Lausanne). 2026 Oct 8;13:1988079. doi: 10.3389/fmed.2026.1988079 (PMC13687000; doi:10.3389/fmed.2026.1988079)
Supplement: Supplementary file 1 [file Datasheet1.pdf]

Supplementary Material

1 Supplementary Data

Figure legends

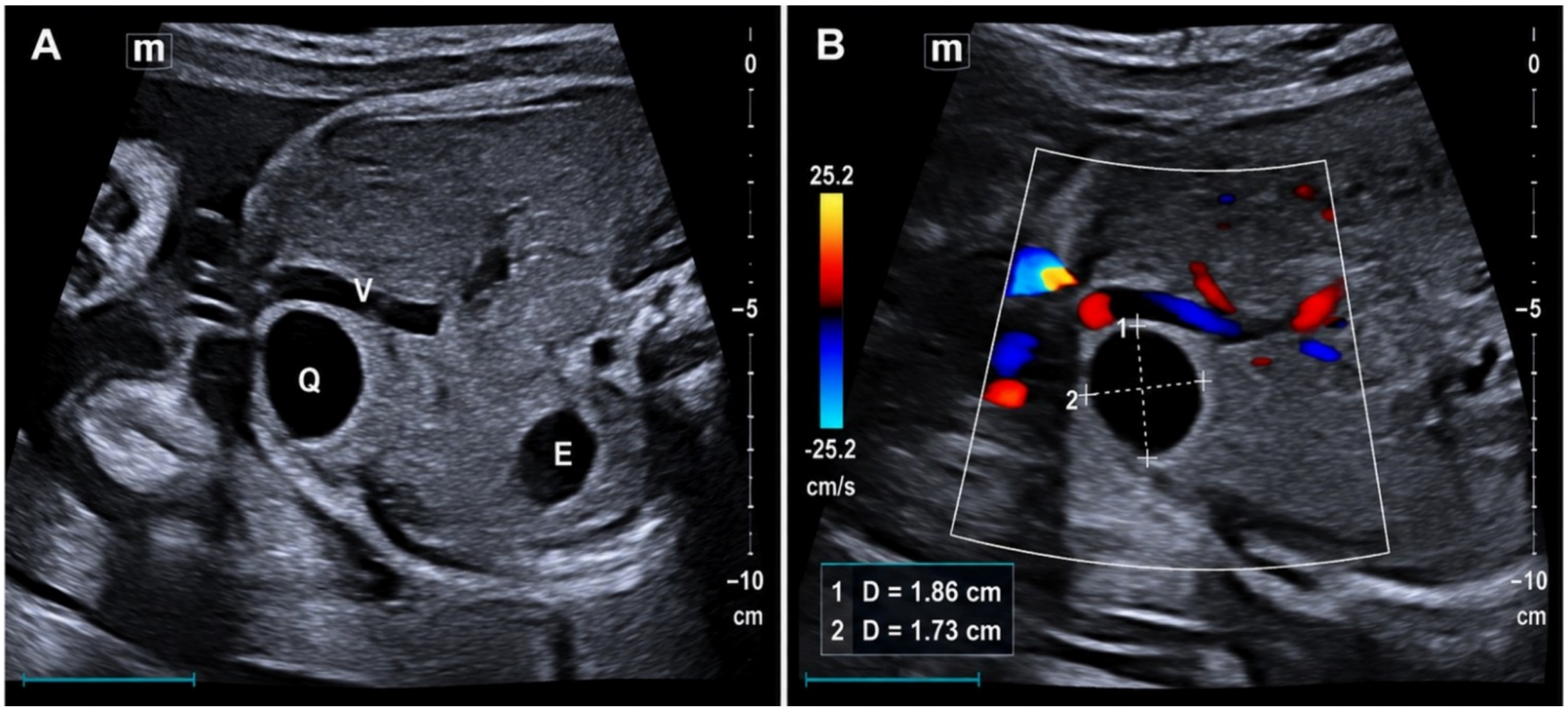

Figure 1. Prenatal ultrasound at 32+3 weeks of gestation. **(A)** Axial grayscale view of the fetal upper abdomen showing a well-defined anechoic cystic lesion (Q) of probable hepatic origin adjacent to the portal venous system (V); the stomach (E) is visualized separately. **(B)** Color Doppler demonstrates the absence of internal vascularity. The lesion measured 18.6 × 17.3 mm.

Alt text – Figure 1: Two-panel prenatal ultrasound at 32+3 weeks of gestation. Panel A shows an axial grayscale view of the fetal upper abdomen with a well-defined, anechoic cystic lesion measuring 18.6 × 17.3 mm adjacent to the portal venous system; the stomach is visualized separately. Panel B shows color Doppler imaging of the same lesion, with no detectable internal vascular flow.

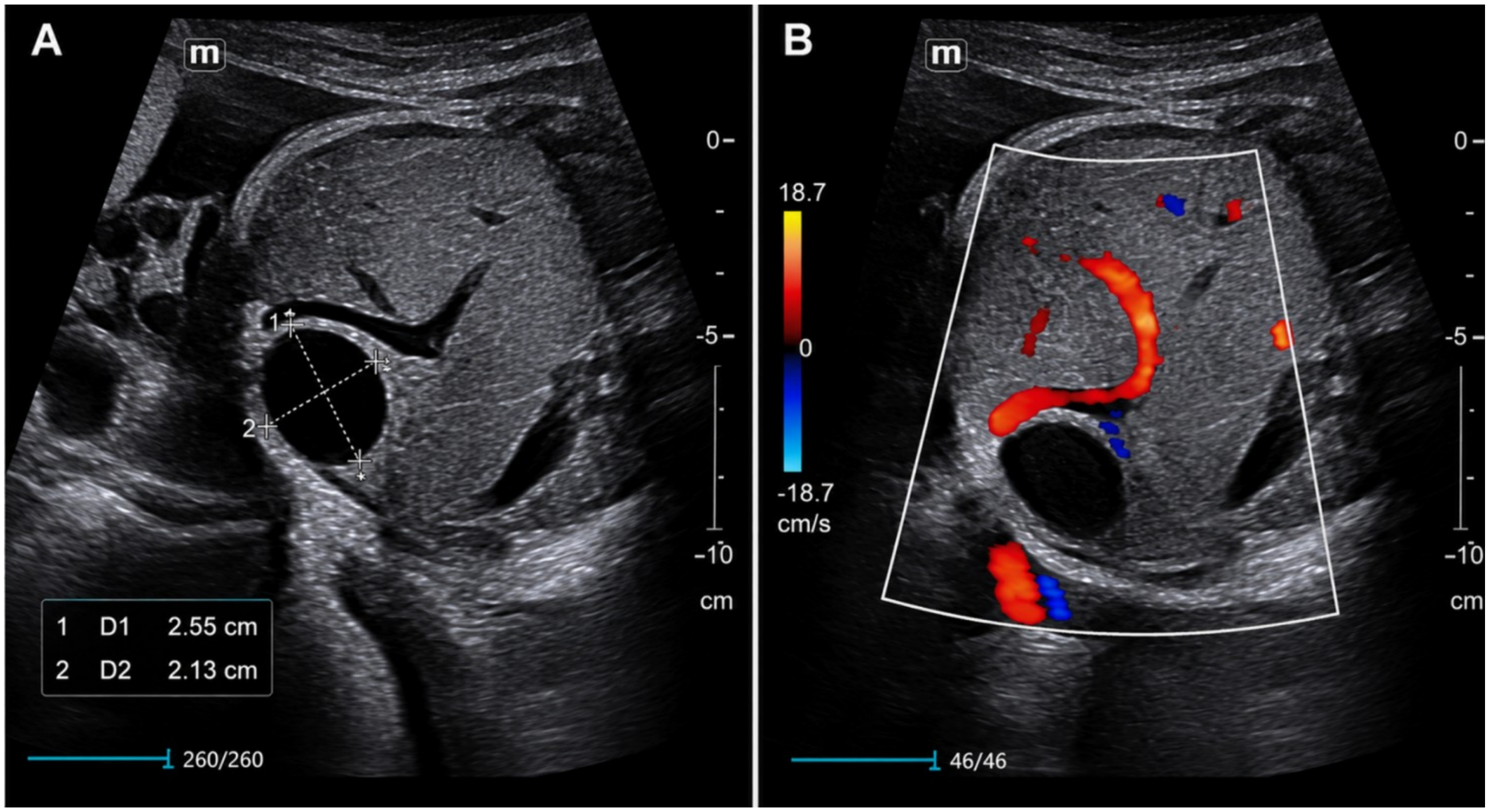

Figure 2. Prenatal ultrasound at 36+3 weeks of gestation. **(A)** Axial grayscale view demonstrating persistence of the well-defined unilocular anechoic cystic lesion, now measuring 25.5 × 21.3 mm. **(B)** Color Doppler confirms the absence of internal vascularity and demonstrates adjacent hepatic and portal vessels.

Alt text – Figure 2: Two-panel prenatal ultrasound at 36+3 weeks of gestation. Panel A shows an axial grayscale view of the fetal upper abdomen with a persistent, well-defined, unilocular anechoic cystic lesion measuring 25.5 × 21.3 mm. Panel B shows color Doppler imaging of the same lesion, demonstrating no detectable internal vascular flow and adjacent hepatic and portal vessels.

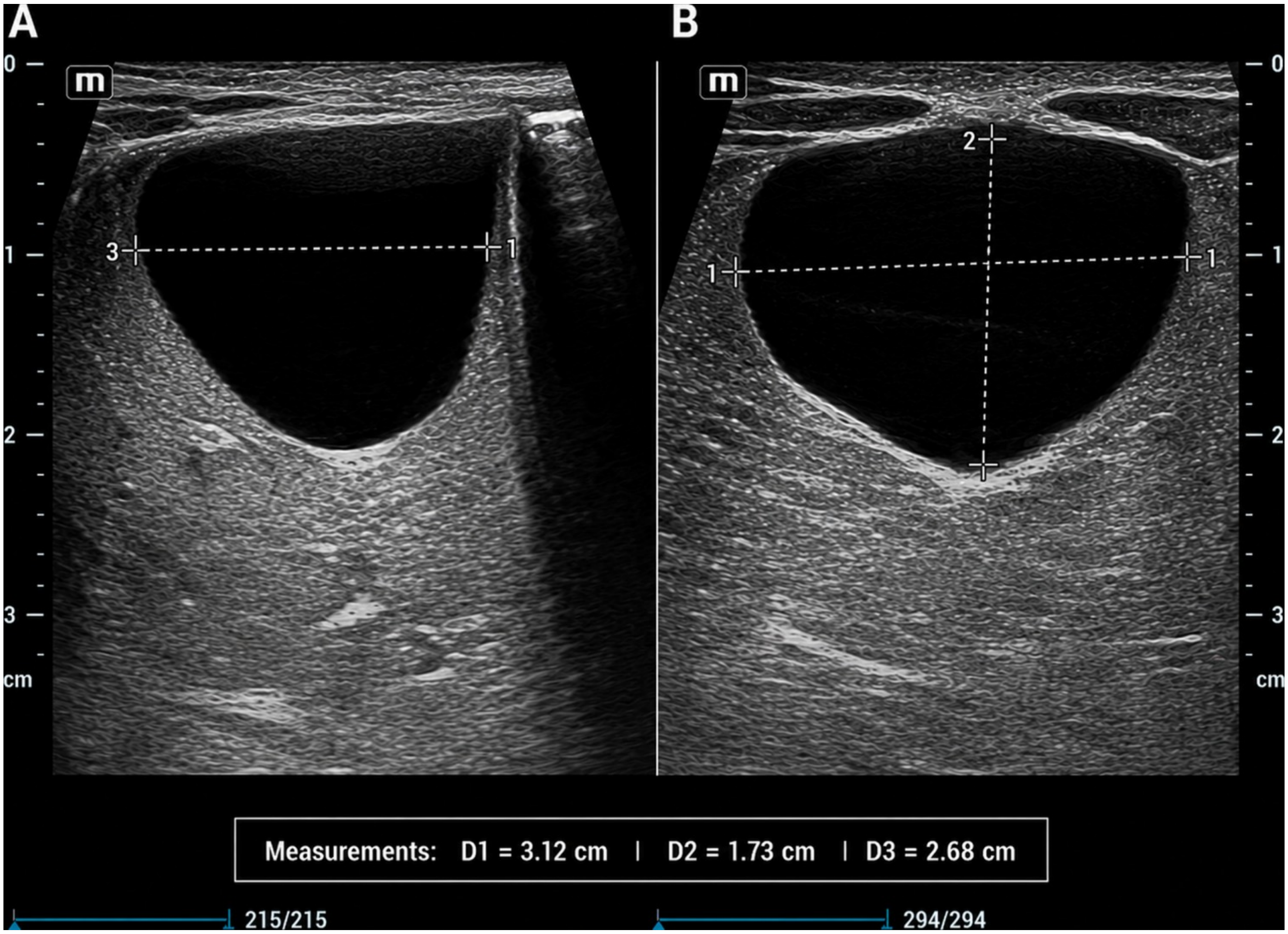

Figure 3. Postnatal abdominal ultrasound on day 3 confirming the congenital simple hepatic cyst. **(A)** Transverse view of the lesion, well defined, unilocular and anechoic, within the hepatic region. **(B)** Orthogonal view demonstrating the maximal transverse and anteroposterior diameters. The three orthogonal diameters obtained across both planes were 31.2 × 17.3 × 26.8 mm. The lesion has thin walls and a simple cystic appearance, with normal intrahepatic and extrahepatic bile ducts.

Alt text – Figure 3: Two-panel postnatal abdominal ultrasound showing a well-defined, unilocular, anechoic cystic lesion in the hepatic region. Panel A demonstrates the lesion in transverse view. Panel B shows an orthogonal view highlighting the maximal transverse and anteroposterior diameters. The three orthogonal diameters measured across both planes were 31.2 × 17.3 × 26.8 mm. The lesion has thin, smooth walls and a simple cystic appearance.

Timepoint	Assessment	Key findings	Clinical management
First trimester	Dating ultrasound (crown–rump length)	Gestational age established; no abnormality identified	Routine antenatal care
Second trimester	Fetal anatomy ultrasound	No cystic lesion identified; fetal growth appropriate	Routine antenatal care
31+2 weeks	Referral third-trimester ultrasound	Unilocular avascular upper-abdominal cyst, 19.3 × 22.1 mm, of probable hepatic origin; severe fetal smallness; AFI ≈4 cm	Serial fetal biometry, amniotic fluid assessment, and Doppler surveillance initiated
32+3 weeks	Follow-up ultrasound (Figure 1)	Persistent avascular cyst, 18.6 × 17.3 mm; oligohydramnios persisted	Continued surveillance
36+3 weeks	Ultrasound and Doppler assessment (Figure 2)	Cyst 25.5 × 21.3 mm, avascular and without mass effect; EFW 1,947 g (<3rd centile); UA PI 60th, MCA PI 99th, and CPR 83rd centile; UA end-diastolic flow present	Stage I late-onset FGR; delivery planned at 37 weeks
37+2 weeks	Elective repeat cesarean delivery	Female neonate, 2,219 g; Apgar scores 7/8; Head circumference 30 cm (<−2 SD for gestational age and sex); left-hand syndactyly with digital hypoplasia	Multidisciplinary neonatal assessment
Postnatal day 1	Neonatal laboratory assessment	Indirect hyperbilirubinemia (total bilirubin 9.5 mg/dL) with ABO incompatibility; direct antiglobulin test negative	Phototherapy for 2 days
Postnatal day 3	Abdominal ultrasonography (Figure 3)	Simple hepatic cyst, 31.2 × 17.3 × 26.8 mm; no biliary dilatation; trace free fluid	Conservative management and discharge
2 months of age	Pediatric and neurological assessment; transfontanellar and abdominal ultrasonography;	Weight 4,200 g; length 53 cm; head circumference 35 cm; normal neurological examination and transfontanellar ultrasonography; Persistent simple anechoic cyst in hepatic segment VIII, 34 × 24 mm,	Continued conservative management with pediatric, imaging, growth, neurodevelopmental, and genetic follow-up

Timepoint	Assessment	Key findings	Clinical management
	laboratory follow-up	without internal vascularity or biliary dilatation; TSH 5.11 μIU/mL within the age-specific reference range; HSV IgM and syphilis testing negative; no cyst-related symptoms	

CARE Checklist (2013) – Completed for Submission

Case Report: Congenital Simple Hepatic Cyst With Late-Onset Fetal Growth Restriction and an Unexpected Neonatal Phenotype

Supplementary Table 1

Completed according to the CARE (CAse REport) reporting guideline. Locations refer to the current manuscript sections and content; final page/line numbers should be updated after typesetting.

CARE item	No.	CARE requirement	Reported in this manuscript	Location / evidence
Title	1	The words "case report" should appear in the title together with the phenomenon of greatest interest.	Yes	Title: "Case Report: Congenital Simple Hepatic Cyst With Late-Onset Fetal Growth Restriction and an Unexpected Neonatal Phenotype."
Keywords	2	2–5 key words that identify diagnoses/interventions and include "case report".	Yes	Keywords include congenital hepatic cyst, fetal growth restriction, prenatal ultrasound, Doppler velocimetry, microcephaly, oligohydramnios, and case report.
Abstract – Introduction	3a	What is unique about this case and what does it add to the medical literature?	Yes	Abstract – Background: rarity of congenital simple hepatic cysts and diagnostic complexity when FGR and additional congenital findings coexist.
Abstract – Main symptoms/findings	3b	Main clinical findings of the patient.	Yes	Abstract – Case presentation: avascular probable hepatic cyst, severe fetal smallness, persistent oligohydramnios; postnatal microcephaly and left-hand syndactyly with digital hypoplasia.
Abstract – Diagnoses/interventions/outcomes	3c	Main diagnoses, therapeutic interventions, and outcomes.	Yes	Abstract – Case presentation: stage I late-onset FGR, delivery at 37+2 weeks, postnatal confirmation of a simple hepatic cyst, conservative management, reassuring 2-month neurological assessment.
Abstract – Conclusion	3d	Main takeaway lesson from the case.	Yes	Abstract – Conclusion: coexistence without established causality; importance of avoiding diagnostic anchoring and maintaining prenatal-to-postnatal etiological reassessment.
Introduction	4	Brief background summary explaining why the case is unique, with references.	Yes	Introduction: congenital hepatic cyst differential diagnosis and management; late-onset FGR diagnosis/staging; rationale and educational value of the case; references 1–10.
Patient information – De-identified demographics	5a	De-identified demographic and other patient-specific information.	Yes	Section 2.1: 31-year-old woman with two previous cesarean deliveries; no identifying personal information.
Patient information – Main concerns/symptoms	5b	Main concerns and symptoms of the patient.	Yes / not symptom-	Section 2.1: referral for routine third-trimester ultrasonography; pregnancy otherwise

			driven	uneventful. The case was detected by imaging rather than maternal symptoms.
Patient information – Medical/family/psychosocial history	5c	Medical, family and psychosocial history, including relevant genetic information.	Yes	Section 2.1: no relevant medical history, maternal comorbidities, smoking/substance use, family history of congenital anomalies, or consanguinity.
Patient information – Relevant past interventions	5d	Relevant previous interventions and outcomes.	Yes	Section 2.1: two previous cesarean deliveries; first-trimester dating and prior anatomy examination documented.
Clinical findings	6	Relevant physical examination and important clinical findings.	Yes	Sections 2.1 and 2.5: normal maternal BP/routine labs; neonatal weight, Apgar scores, head circumference <−2 SD, left-hand syndactyly with digital hypoplasia, and otherwise reassuring examination.
Timeline	7	Important dates and times in the case organized as a timeline.	Yes	Section 2.7, Table 1: first trimester, second trimester, 31+2, 32+3, 36+3, 37+2 weeks, postnatal days 1 and 3, and 2-month follow-up.
Diagnostic assessment – Methods	8a	Diagnostic methods, including examination, laboratory testing, imaging, surveys, etc.	Yes	Sections 2.1–2.3 and 2.5–2.6: maternal serology; serial fetal ultrasound; color/pulsed Doppler; fetal biometry; AFI; UA/MCA/DV/uterine artery Doppler; computerized CTG; biophysical assessment; neonatal and follow-up ultrasonography.
Diagnostic assessment – Challenges	8b	Diagnostic challenges such as access, financial, cultural or other barriers.	Partially / clinically addressed	Section 2.2: prenatal uncertainty regarding exact origin of the upper-abdominal cyst and differential diagnosis. Section 2.5: cross-sectional imaging was not performed because ultrasound findings were considered unequivocal and the infant was asymptomatic. Definitive genetic testing was not performed and is acknowledged as a limitation.
Diagnostic assessment – Diagnosis/differential	8c	Diagnostic reasoning, including differential diagnoses considered.	Yes	Section 2.2: probable hepatic origin; choledochal, mesenteric, enteric duplication, and ovarian cysts considered. Section 2.3: Delphi late-onset FGR and Barcelona stage I classification. Discussion revisits developmental/genetic differential after neonatal phenotype.
Diagnostic assessment – Prognostic characteristics	8d	Prognostic characteristics when applicable.	Yes	Section 2.3: EFW <3rd percentile, UA PI 60th, MCA PI 99th, CPR 83rd percentile, preserved UA end-diastolic flow, normal DV, reassuring CTG/biophysical assessment, persistent oligohydramnios.
Therapeutic intervention – Type	9a	Types of therapeutic intervention, including preventive/self-care if relevant.	Yes	Sections 2.4–2.6: serial surveillance, planned delivery at 37+2 weeks, elective repeat cesarean for obstetric history, neonatal phototherapy for indirect hyperbilirubinemia, conservative cyst management.
Therapeutic intervention – Administration	9b	Administration of intervention, including dose, strength and duration when applicable.	Yes where applicable	Section 2.5: phototherapy for 2 days. Obstetric intervention is described by timing and mode; drug dosing is not applicable.
Therapeutic intervention – Changes	9c	Changes in interventions with rationale.	Yes / no major change required	Serial surveillance continued because Doppler and fetal testing remained reassuring; uncomplicated cyst did not alter delivery timing. Conservative management continued because there was no mass effect, biliary involvement, or symptoms.
Follow-up and outcomes – Clinician/patient outcomes	10a	Clinician- and patient-assessed outcomes when appropriate.	Yes	Section 2.6: clinically well at 2 months; weight 4,200 g, length 53 cm, head circumference 35 cm; normal neurological examination and transfontanellar ultrasound; persistent uncomplicated hepatic cyst.
Follow-up and outcomes – Follow-up tests	10b	Important follow-up diagnostic and other test results.	Yes	Section 2.6: abdominal ultrasound showed persistent simple cyst in segment VIII without vascularity or biliary dilatation; TSH within age-specific range; HSV IgM and syphilis testing negative.
Follow-up and outcomes – Adherence/tolerability	10c	Intervention adherence and tolerability, including how assessed.	Not applicable / indirectly reported	No long-term medication regimen was prescribed. Infant remained asymptomatic with normal feeding and bowel habits; family agreed to continued pediatric, imaging and genetic follow-up.
Follow-up and outcomes –	10d	Adverse and unanticipated	Yes	Section 2.5: indirect hyperbilirubinemia with

Adverse/unanticipated events		events.		ABO incompatibility treated with phototherapy. Unexpected neonatal phenotype included microcephaly and left-hand syndactyly with digital hypoplasia.
Discussion – Strengths/limitations	11a	Strengths and limitations of the approach to the case.	Yes	Discussion: longitudinal prenatal-to-postnatal characterization and multidisciplinary follow-up listed as strengths; absence of histopathology, cross-sectional imaging, definitive genetic testing, placental histopathology, and long-term follow-up listed as limitations.
Discussion – Relevant literature	11b	Discussion of relevant medical literature.	Yes	Discussion integrates congenital hepatic cyst literature, Delphi FGR definition, Barcelona stage-based protocol, and ISUOG guidance.
Discussion – Rationale for conclusions	11c	Scientific rationale for conclusions, including possible causes.	Yes	Discussion explicitly avoids causal attribution between cyst and FGR; considers developmental/genetic contribution and explains that neonatal phenotype broadens rather than invalidates the prenatal FGR diagnosis.
Discussion – Take-away lesson	11d	Primary take-away lesson from the case.	Yes	Discussion and Conclusion: avoid diagnostic anchoring; maintain prenatal-to-postnatal reassessment when severe fetal smallness coexists with structural or newly identified congenital abnormalities.
Patient perspective	12	Patient should share perspective or experience whenever possible.	Yes	Section 4 Patient Perspective: maternal concern, counseling regarding cyst/FGR, surveillance and delivery planning, postnatal confirmation, and agreement with continued follow-up.
Informed consent	13	Was informed consent obtained? Provide if requested.	Yes	Ethics statement: written informed consent obtained from the mother for publication of her clinical information and the child's clinical information and images; identifying information removed.

Reference: Gagnier JJ, Kienle G, Altman DG, Moher D, Sox H, Riley D; CARE Group. The CARE guidelines: consensus-based clinical case reporting guideline development. BMJ Case Rep. 2013;2013:bcr2013201554. doi:10.1136/bcr-2013-201554.
